# Cognitive subgroups in bipolar disorder: associations with brain-derived neurotrophic factor and C-reactive protein

**DOI:** 10.1192/bjo.2025.10918

**Published:** 2025-12-10

**Authors:** Ahmet Mete Demir, Yasemin Görgülü, Kübra Söğüt, Orkide Palabıyık, Elif Ayduk Gövdeli

**Affiliations:** Liaison Psychiatry, Derbyshire Healthcare NHS Foundation Trust, Chesterfield Royal Hospital, Chesterfield, UK; Department of Psychiatry, Faculty of Medicine, Trakya University, Edirne, Turkey; Health Services Vocational College, Trakya University, Edirne, Turkey; Cardiovascular Magnetic Resonance (CMR) Unit, Guy’s and St Thomas’ NHS Foundation Trust, Royal Brompton Hospital, London, UK

**Keywords:** Bipolar disorder, cognitive subgroups, BDNF, CRP

## Abstract

**Background:**

Cognitive impairment varies widely in bipolar disorder. Identifying cognitive subgroups and their biological correlates may improve understanding of the disorder. Brain-derived neurotrophic factor (BDNF) and C-reactive protein (CRP) are key candidates due to their roles in neuroplasticity and inflammation.

**Aims:**

The aim was to investigate cognitive subgroups in patients with bipolar disorder and their association with serum levels of BDNF and CRP.

**Method:**

A cross-sectional study was conducted on 149 bipolar disorder patients and 48 healthy controls. Cognitive performance was assessed using a comprehensive battery of neuropsychological tests. Cluster analysis was performed to identify cognitive subgroups, followed by discriminant function analysis to validate the classification. Serum levels of BDNF and CRP were measured and compared across cognitive subgroups.

**Results:**

Cluster analysis identified three cognitive subgroups: intact cognition, selectively impaired cognition (SIC) and globally impaired cognition (GIC). SIC exhibited the highest BDNF levels, while GIC demonstrated the highest CRP levels. CRP levels were negatively associated with performance across all cognitive domains. BDNF showed a negative correlation with verbal fluency, short-term memory and working memory. CRP levels exceeding 4.3 mg/L predicted global cognitive impairment with a sensitivity of 72.41% and specificity of 73.63%.

**Conclusion:**

Cognitive impairments in bipolar disorder patients can be classified into distinct subgroups, which are associated with serum levels of BDNF and CRP. These findings suggest that inflammation and neuroplasticity play key roles in the pathophysiology of cognitive decline in bipolar disorder, providing potential biomarkers for identifying patients at risk for severe cognitive impairments.

Cognitive deficits, particularly in executive function, are commonly observed in bipolar disorder, both during acute phases and in euthymic states.^1,2^ These deficits are not merely transient but often persist long after mood symptoms subside, significantly affecting individuals’ functional outcomes and overall quality of life.^3^ Research suggests that cognitive impairments in bipolar disorder can manifest across a wide range of domains, including attention, processing speed, working memory and verbal fluency, all of which contribute to challenges in daily living and occupational performance.^4,5^ The persistence of these impairments highlights the importance of understanding their underlying mechanisms and identifying potential markers for early detection and intervention.

An emerging area of interest in bipolar disorder research is the concept of cognitive heterogeneity. Recent studies indicate that cognitive impairments in bipolar disorder do not affect all individuals uniformly. Instead, discrete cognitive subgroups can be identified, each characterised by varying degrees of impairment in different cognitive domains.^6,7^ This cognitive heterogeneity suggests that bipolar disorder may encompass a spectrum of neurocognitive presentations, potentially linked to different pathophysiological mechanisms. In a study involving 136 patients with bipolar disorder, three distinct cognitive subgroups were identified: one with relatively intact cognitive function, a second with selective impairments in specific domains (such as verbal memory or executive function) and a third with more global cognitive impairments across multiple domains.^8^ A recent meta-analysis further confirmed this three-subtype structure, providing strong evidence for the reproducibility of severe-impairment, mild-impairment and good-performance groups in bipolar disorder.^9^ This growing body of evidence provides a rationale for examining potential biological markers, such as brain-derived neurotrophic factor (BDNF) and C-reactive protein (CRP), which may help differentiate these cognitive subgroups.

Understanding the biological underpinnings of these cognitive subgroups has become a key focus in recent bipolar disorder research. Biomarkers such as BDNF and CRP are of particular interest because of their established roles in neuroplasticity and inflammation, respectively. BDNF is a neurotrophin essential for synaptic plasticity and cognitive function and altered BDNF levels have been linked to cognitive impairments in bipolar disorder and various neuropsychiatric conditions.^10^ For instance, low serum BDNF levels have been associated with poorer cognitive performance in domains such as verbal memory and executive function, particularly during euthymic periods of bipolar disorder.^11^ Furthermore, research suggests that BDNF levels may fluctuate depending on mood states, with higher levels observed during manic or depressive episodes and lower levels during remission, indicating a complex relationship between BDNF and cognitive function.^12,13^

CRP, on the other hand, is a widely recognised marker of systemic inflammation. Elevated CRP levels have been associated with cognitive decline in both psychiatric and non-psychiatric populations.^14,15^ Inflammation has long been implicated in the pathophysiology of bipolar disorder, and increasing evidence suggests that inflammatory processes may contribute to cognitive impairments in this population.^16^ A recent meta-analysis demonstrated a robust association between elevated inflammatory markers, including CRP, and cognitive deficits in individuals with bipolar disorder, suggesting that inflammation may play a mediating role in the observed cognitive decline.^17^ The relationship between CRP and cognitive function in bipolar disorder is particularly intriguing, as it may provide insights into the role of neuroinflammation in the neuroprogressive course of the disorder.

Given these associations, it is plausible that variations in the serum levels of BDNF and CRP may correspond to differences in cognitive profiles among individuals with bipolar disorder. By examining these biomarkers within well-defined cognitive subgroups, we aim to determine whether BDNF and CRP levels can serve as potential indicators of cognitive impairment severity or subgroup classification. Understanding how these biological markers are associated with cognitive outcomes in bipolar disorder is important for establishing their potential as measurable indicators of cognitive decline, and may provide a foundation for future biomarker-informed treatment approaches. Recent advances in the study of bipolar disorder biomarkers underscore the importance of exploring these relationships further, as they hold promise for enhancing our ability to predict and treat cognitive impairments in this population.

In this study, we investigated the clinical and biological differences among cognitive subgroups in patients with bipolar disorder, focusing on the relationship between serum levels of BDNF and CRP. Specifically, we seek to characterise the cognitive subgroups, explore the association between these biomarkers and cognitive performance and assess whether BDNF and CRP can be used to differentiate between cognitive subgroups. By elucidating these relationships, we hope to contribute to the growing body of literature on cognitive heterogeneity in bipolar disorder and provide insights into the development of more personalised treatment approaches based on cognitive profiles and biomarker levels.

## Method

Our study at Trakya University Faculty of Medicine Psychiatry Department employed a cross-sectional design, spanning from March 2021 to March 2022, to examine the relationship between cognitive subgroups and serum levels of BDNF and CRP in patients diagnosed with bipolar disorder. Cognitive assessments and blood samples were collected on the same day for each participant to ensure the reliability of data collection and to minimise confounding factors related to mood variability.

A total of 149 patients, aged between 18 and 65 years, with a confirmed diagnosis of bipolar disorder-I based on DSM-5^18^ criteria, and 48 age- and sex-matched healthy controls were included in the study. Participants were required to have been in a euthymic state for at least three months at the time of assessment, as defined by a score of ≤7 on both the Hamilton Depression Rating Scale (HDRS)^19^ and the Young Mania Rating Scale (YMRS).^20^ Patients with psychotic symptoms, schizoaffective disorder, schizophrenia or comorbid psychiatric conditions such as anxiety disorders, alcohol or substance use disorders, as well as those with a history of neurological disorders, stroke, learning disability, autoimmune illnesses, acute infections, malignancies, severe head trauma and a history of electroconvulsive therapy within the past year, were excluded from the study. The sample consisted of 107 women and 90 men, reflecting a gender-balanced population.

Cognitive function was assessed using a battery of standardised neuropsychological tests designed to evaluate various cognitive domains. The primary tools used for cognitive evaluation were the Stroop Test^21^ for processing speed and inhibitory control, the Rey Auditory Verbal Learning Test (RAVLT)^22^ for short- and long-term verbal memory, the Controlled Oral Word Association Test (COWAT)^23^ for verbal fluency and the Digit Span Test (DST)^24^ for working memory, respectively. Additionally, the Clock Drawing Test^25^ was conducted to assess and rule out potential dementia. These tests were selected based on their widespread use in psychiatric research and their established validity and reliability in assessing cognitive function. Patient performance on each test was measured and standardised against scores from healthy controls, enabling direct comparison across groups. The results were subsequently used to categorise participants into different cognitive subgroups based on their relative performance across the assessed domains.

Blood samples were collected from each patient between 08.00 h and 10.30 h following an overnight fast, concurrent with cognitive testing, to control for potential diurnal variations in BDNF and CRP levels. The samples were left at room temperature for 30 min before being centrifuged at 3000 rpm for 10 min at +4 °C at the Trakya University Biophysics Laboratory. The resulting serum samples were then divided into Eppendorf tubes and stored in a deep freezer at −80 °C until use with an ELISA kit for BDNF (Invitrogen, Catalogue: EH42RB), according to the manufacturer’s instructions. CRP levels were also measured in the laboratory. In addition, patients with bipolar disorder were assessed for drug serum (lithium, valproate) and thyroid-stimulating hormone (TSH) levels.

Informed written consent was obtained from all participants prior to their inclusion in the study. The authors assert that all procedures contributing to this work comply with the ethical standards of the relevant national and institutional committees on human experimentation and with the Helsinki Declaration of 1975, as revised in 2013. All procedures involving human participants were approved by the Trakya University Faculty of Medicine Ethics Committee on 1 March 2021 with approval number TUTF-BAEK 2021/108.

### Statistical analysis

Data were analysed using IBM SPSS Statistics for Windows, version 20.0 (License No: 10240642, IBM, Armonk, New York, USA; see https://www.ibm.com/products/spss). Parametric continuous data were expressed as mean ± s.d., non-parametric continuous data as median (minimum–maximum) and categorical data as percentages. Normal distribution was assessed using the Kolmogorov–Smirnov test. Neuropsychological battery results were transformed into *z*-scores, with a *z*-score > −1.5 indicating mild cognitive impairment. This cut-off was chosen as it offers an optimal balance between sensitivity and specificity in detecting impairment, while also allowing comparability with previous studies on mild cognitive impairment.^26,27^ The *z*-scores lower than −4.0 were truncated to −4.0 for inclusion. We examined the potential cognitive subgroups among patients using Ward’s method, followed by the K-means method. This classification served as the basis for analysing the relationship between cognitive profiles and biomarker levels. Group classification was then verified by discriminant function analysis (DFA). The Wilks’ Lambda (*λ*) test was performed to determine the extent to which each function categorised the patients into subgroups. A multivariate analysis of variance was performed to test whether different cognitive domains showed significant group differences.

Between-group comparisons were performed using Pearson’s chi-square, analysis of variance or Kruskal–Wallis tests, followed by Tukey’s honest significant difference or Dunn’s test with Bonferroni correction, according to the normality of the data. Spearman’s correlation test was used to explore the associations between serum BDNF and CRP levels and cognitive domains. Multicollinearity among independent variables was assessed using Pearson correlation coefficients and variance inflation factor (VIF) values. Logistic regression was used to analyse parameters affecting cognitive impairment, and receiver operating characteristic (ROC) curve analysis was used to determine cut-off values for impairment. The value *p* < 0.05 was considered statistically significant for all analyses.

## Results

The cluster analysis revealed that the 149 patients with bipolar disorder were most effectively grouped into three distinct clusters. The first cluster, with intact cognitive functions, included 53 individuals (35.6%), while the second cluster, showing mild to moderate impairments across several cognitive domains (SIC), comprised 38 individuals (25.5%) and the third cluster, characterised by severe impairments in all cognitive domains (GIC), consisted of 58 individuals (38.9%). The comparison of the *z*-scores of cognitive subgroups across distinct cognitive domains is presented in Table 1.

**Table 1:** The comparison of the *z*-scores of cognitive subgroups in bipolar disorder across distinct cognitive domains

	IC (*n* = 53)	SIC (*n* = 38)	GIC (*n* = 58)	*p*
Inhibition control	−0.48 ± 1.09^b^	−0.10 ± 0.92^c^	−2.92 ± 1.09^b^ ^c^	<0.001***
Processing speed	−0.68 ± 1.02^b^	−0.94 ± 1.12^c^	−3.52 ± 0.62^b^ ^c^	<0.001***
Verbal fluency	−0.73 ± 1.12^a^ ^b^	−2.41 ± 0.89^a^	−2.46 ± 1.06^b^	<0.001***
Short-term verbal memory	−0.53 ± 0.89^a^ ^b^	−1.82 ± 0.72^a^	−2.16 ± 0.58^b^	<0.001***
Long-term verbal memory	−0.64 ± 1.10^a^ ^b^	−3.29 ± 0.94^a^	−3.17 ± 0.90^b^	<0.001***
Working memory	−0.78 ± 0.78^a^ ^b^	−1.34 ± 0.78^a^ ^c^	−2.11 ± 0.55^b^ ^c^	0.001**

Multivariate analysis of variance test and Tukey’s honest significant difference test.

IC, intact cognition; SIC, selectively impaired cognition; GIC, globally impaired cognition.

a. Statistically significant difference between IC and SIC groups.

b. Statistically significant difference between IC and GIC groups.

c. Statistically significant difference between SIC and GIC groups.

**p < 0.01, ***p < 0.001.

The accuracy of this grouping was verified using DFA, which identified two discriminant functions. The first discriminant function (Wilks’ *λ* = 0.10; *χ* ^2^ = 326.60; *p* < 0.001) accounted for 76.7% of the variance between the groups, while the second function (Wilks’ *λ* = 0.47; *χ* ^2^ = 106.68; *p* < 0.001) explained the remaining 23.3% of the variance. Using these functions, 98% of the patients were correctly classified into their respective clusters, confirming the validity of the three neurocognitive subgroups. Long-term and short-term verbal memory, along with processing speed, contributed the most to the clustering process. The standardised canonical discriminant coefficients were 0.56 for long-term verbal memory, 0.35 for short-term verbal memory and 0.39 for processing speed.

The average age of the study participants was 42.12 ± 11.9 years, with 45.7% of the patients (90 individuals) being male. The mean ages of patients in the SIC and GIC groups were significantly higher than those in the intact cognition and healthy control groups (46.7 ± 12.1 years and 46.5 ± 10.7 years versus 36.3 ± 10.4 years and 39.7 ± 11.5 years, respectively; *p* < 0.001). No significant differences were found in the sex distribution across the groups.

Educational status differed significantly between the groups, with individuals possessing only a primary school education being more prevalent in the SIC and GIC groups. Conversely, the proportion of individuals with a university education was notably higher in the healthy control group, alongside a higher employment rate.

No significant differences were observed between the groups in terms of age at illness onset, duration of remission, total hospital admissions, the duration of valproate use, valproate levels, TSH levels, SSRI use, antipsychotic use and antipsychotic type or number of episodes when evaluating the progression of the illness. However, the intact cognition group showed a significantly shorter total illness duration compared with the other groups (144.36 ± 98.76 weeks versus 216.16 ± 147.22 weeks and 234.10 ± 143.38 weeks; *p* = 0.001). Furthermore, the duration of lithium use was significantly lower in the intact cognition group compared with the GIC group (42 (8–300) months versus 132 (10–480) months).

The lithium levels (mmol/L) differed significantly among the cognitive subgroups (*p* = 0.046). The mean (± s.d.) lithium levels were 0.59 ± 0.18 in the intact cognition group, 0.64 ± 0.15 in the SIC group and 0.72 ± 0.22 in the GIC group. Following statistical analysis, the difference in lithium levels between the intact cognition and GIC groups remained significant (*p* = 0.035), while the differences between the intact cognition and SIC groups (*p* = 0.719) and the SIC and GIC groups (*p* = 0.315) were not statistically significant. Additional characteristic features of the participants are presented in Table 2.

**Table 2:** Sociodemographic and clinical characteristics of neurocognitive subgroups and demographic characteristics of healthy controls

	IC (*n* = 53)	SIC (*n* = 38)	GIC (*n* = 58)	HC (*n* = 48)	*p*
Age (years)	36.3 ± 10.4^a^ ^b^	46.7 ± 12.1^a^ ^e^	46.5 ± 10.7^b^ ^f^	39.7 ± 11.5^e^ ^f^	<0.001***
BMI (kg/m^2^)	27.9 ± 5.3^a^ ^b^ ^c^	30.7 ± 4.7^a^ ^e^	30.0 ± 5.4^b^ ^f^	25.6 ± 3.8^c^ ^e^ ^f^	<0.001***
Male gender (*n*, %)	22 (41.5)	19 (50)	26 (44.8)	23 (47.9)	0.856
Education					
Primary school (*n*, %)	5 (9.4)	19 (50)	35 (60.4)	1 (2.1)	<0.001***
High school (*n*, %)	22 (41.5)	11 (28.9)	18 (31)	14 (29.2)	0.486
University (*n*, %)	26 (49.1)	8 (21.1)	5 (8.6%)	33 (68.8)	<0.001***
Employment					
Employed (*n*, %)	24 (45.3)	10 (26.3)	17 (29.3)	42 (87.5)	<0.001***
Marital status					
Married (*n*, %)	19 (35.8)	23 (60.5)	38 (65.5)	33 (68.8)	0.003**
Family history					
Anxiety disorders (*n*, %)	3 (11.5)	0 (0%)	2 (8)	7 (41.2)	0.002**
Mood disorders (*n*, %)	17 (65.4)	13 (61.9)	18 (72)	4 (23.5)	0.011*
Psychotic disorders (*n*, %)	6 (23.1)	7 (33.3)	5 (20)	3 (17.6)	0.655
Others (*n*, %)	0 (0)	1 (4.8)	0 (0)	3 (17.6)	0.026*
Clinical characteristics					
Age at onset (years)	24.0 ± 7.77^a^	28.63 ± 9.95^a^	26.79 ± 9.55	–	0.051
Total illness duration (months)	144.36 ± 98.76^a^ ^b^	216.16 ± 147.22^a^	234.10 ± 143.38^b^	–	0.001**
Remission duration (months)	10 (3–192)	24 (3–120)	12 (3–180)	–	0.440
Number of hospital admissions (*n*)	3 (0–16)	2 (0–10)	2 (0–14)	–	0.527
Number of manic episodes (*n*)	2 (1–11)	3 (1–20)	3 (1–25)	–	0.063
Number of depressive episodes (*n*)	2 (0–15)	2 (0–10)	2 (0–20)	–	0.638
Lithium level (mmol/L)	0.59 ± 0.18^b^	0.64 ± 0.15	0.72 ± 0.22^b^	–	0.046*
Total lithium use duration (months)	42 (8–300)^b^	102 (3–360)	132 (10–480)^b^	–	0.003**
Valproate level (mg/L)	67 (17–106)	60 (41–118)	63.5 (38–93)	–	0.956
Total valproate use duration (months)	48 (2–204)	48 (5–348)	60 (12–192)	–	0.658
TSH level (μIU/ml)	2.2 (0.1–6.4)	2.1 (0.2–12.2)	1.8 (0.1–10)	–	0.510
SSRI use (*n*, %)	3 (5.7)	11 (28.9)	6 (10.3)	–	0.060
Antipsychotic use (*n*, %)					
Typical	0 (0)	1 (2.6)	0 (0)	–	0.088
Atypical	46 (86.8)	30 (78.9)	46 (79.3)		
Typical and atypical	6 (11.3)	1 (2.6)	7 (12.1)		
None	1 (1.9)	6 (15.8)	5 (8.6)		

Chi-square, analysis of variance and Kruskal–Wallis tests, followed by Tukey’s honest significant difference test and Dunn’s test with Bonferroni correction.

IC, intact cognition; SIC, selectively impaired cognition; GIC, globally impaired cognition; HC, healthy controls; BMI, body mass index; TSH, thyroid-stimulating hormone; SSRI, selective serotonin reuptake inhibitor.

a. Statistically significant difference between IC and SIC groups.

b. Statistically significant difference between IC and GIC groups.

c. Statistically significant difference between IC and HC groups.

d. Statistically significant difference between SIC and GIC groups.

e. Statistically significant difference between SIC and HC groups.

f. Statistically significant difference between GIC and HC groups.

*p < 0.05, **p < 0.01, ***p < 0.001.

The intact cognition group exhibited superior performance on neurocognitive tests compared with the other two cognitive subgroups. Specifically, the intact cognition group performed better on the Stroop test, showing no significant deficits in executive function or processing speed. However, significant impairments in executive function, particularly inhibition control, were observed in both the SIC and GIC groups. The intact cognition group also outperformed the SIC and GIC groups on other neurocognitive assessments, including the RAVLT, COWAT and DST. A detailed comparison of neurocognitive test performance across the groups is presented in Table 3.

**Table 3:** Neurocognitive test results of cognitive subgroups and healthy controls

	IC (*n* = 53)	SIC (*n* = 38)	GIC (*n* = 58)	HC (*n* = 48)	*p*
Clock drawing test score	10 (5–10)^b^	10 (5–10)	10 (5–10)^b^ ^f^	10 (9–10)^f^	<0.001***
Inhibitory control and processing speed – Stroop test					
Word-reading trial					
Total duration (s)	9 (6–58)^b^	11 (5–20)^e^	12 (8–29)^b^	9 (6–13)^e^	<0.001***
Number of corrections (*n*)	0 (0–1)	0 (0–2)	0 (0–1)	0 (0–0)	0.151
Number of errors (*n*)	0 (0–2)	0 (0–0)	0 (0–2)	0 (0–0)	0.367
Colour naming trial					
Total duration (s)	17 (13–30)^b^ ^c^	19 (11–51)^d^ ^e^	28 (16–56)^b^ ^d^ ^f^	13 (9–23)^c^ ^e^ ^f^	<0.001***
Number of corrections (*n*)	0 (0–2)	0 (0–4)^d^	0 (0–5)^d^ ^f^	0 (0–2)^f^	<0.001***
Number of errors (*n*)	0 (0–1)^b^	0 (0–1)	0 (0–3)^b^ ^f^	0 (0–1)^f^	0.002**
Interference trial					
Total duration (s)	27 (15–42)^b^	26.5 (13–44)^d^	44 (27–101)^b^ ^d^ ^f^	22 (12–39)^f^	<0.001***
Number of corrections (*n*)	0 (0–4)^b^	0 (0–5)^d^	2 (0–4)^b^ ^d^ ^f^	0 (0–3)^f^	0.001**
Number of errors (*n*)	0 (0–10)^b^	0 (0–5)^d^	1 (0–8)^b^ ^d^ ^f^	0 (0–4)^f^	<0.001***
Verbal fluency – COWAT					
Number of words (*n*)	55 (21–86)^a^ ^b^	27 (8–57)^a^ ^e^	25.5 (4–76)^b^ ^f^	65 (32–95)^e^ ^f^	<0.001***
Number of perseverations (*n*)	0 (0–3)	0 (0–6)	0 (0–5)	0 (0–2)	0.081
Short-term and long-term verbal memory – RAVLT					
Immediate recall score	8 (4–14)^a^ ^b^	6 (2–10)^a^ ^e^	5 (1–10)^b^ ^f^	10 (5–15)^e^ ^f^	<0.001***
Total learning score	138 (95–149)^a^ ^b^	119.5 (58–146)^a^ ^e^	100 (49–137)^b^ ^f^	144.5 (94–150)^e^ ^f^	<0.001***
Delayed recall score	13 (10–15)^a^ ^b^	9 (4–13)^a^ ^e^	9 (2–13)^b^ ^f^	15 (11–15)^e^ ^f^	<0.001***
Working memory – DST					
Forwards score	5 (4–9)^b^ ^c^	5 (4–9)^e^	5 (4–7)^b^ ^f^	7 (4–9)^c^ ^e^ ^f^	<0.001***
Backwards score	4 (3–6)^a^ ^b^ ^c^	4 (2–6)^a^ ^e^	3 (2–5)^b^ ^f^	5 (3–8)^c^ ^e^ ^f^	<0.001***

Kruskal–Wallis test, followed by Dunn’s test with Bonferroni correction. Values presented in brackets are raw numerical scores produced by the assessment.

IC, intact cognition; SIC, selectively impaired cognition; GIC, globally impaired cognition; HC, healthy controls; COWAT, controlled oral word association test; RAVLT, Rey auditory verbal learning test; DST, digit span test.

a. Statistically significant difference between IC and SIC groups.

b. Statistically significant difference between IC and GIC groups.

c. Statistically significant difference between IC and HC groups.

d. Statistically significant difference between SIC and GIC groups.

e. Statistically significant difference between SIC and HC groups.

f. Statistically significant difference between GIC and HC groups.

**p < 0.01, ***p < 0.001.

The SIC group demonstrated the highest levels of BDNF compared with the intact cognition and GIC groups, with a statistically significant difference (*p* < 0.001). In contrast, the GIC group exhibited the highest levels of CRP compared with the intact cognition and SIC groups, also with significant differences (*p* < 0.001). These results are presented in Table 4.

**Table 4:** Serum BDNF and CRP levels of cognitive subgroups

	IC (*N* = 53)	SIC (*N* = 38)	GIC (*N* = 58)	*p*
BDNF levels (ng/ml)	21.71 ± 12.31^a^	38.15 ± 16.71^a^ ^c^	25.73 ± 17.36^c^	<0.001***
CRP levels (mg/L)	3.91 ± 2.70^b^	3.93 ± 3.15^c^	6.94 ± 4.48^b^ ^c^	<0.001***

Analysis of variance and Tukey’s honest significant difference test.

IC, intact cognition; SIC, selectively impaired cognition; GIC, globally impaired cognition; BDNF, brain-derived neurotrophic factor; CRP, C-reactive protein.

a. Statistically significant difference between IC and SIC groups.

b. Statistically significant difference between IC and GIC groups.

c. Statistically significant difference between SIC and GIC groups.

***p < 0.001.

Further analysis revealed a negative correlation between CRP levels and performance across all neurocognitive domains, suggesting that higher inflammation levels were associated with greater cognitive deficits. Conversely, BDNF levels were negatively correlated with performance in verbal fluency, short-term memory, long-term memory and working memory domains (Table 5).

**Table 5:** Correlation of BDNF and CRP with cognitive domains

	BDNF	CRP
Inhibitory control		
rho	0.190	−0.404
* p*	0.021*	<0.001***
Processing speed		
rho	0.097	−0.458
* p*	0.239	<0.001***
Verbal fluency		
rho	−0.431	−0.256
* p*	<0.001***	0.002**
Verbal short-term memory		
rho	−0.306	−0.284
* p*	0.003**	<0.001***
Verbal long-term memory		
rho	−0.468	−0.350
* p*	<0.001***	0.001**
Working memory		
rho	−0.239	−0.288
* p*	0.001**	<0.001***

Spearman’s correlation.

BDNF, brain-derived neurotrophic factor; CRP, c-reactive protein.

*p < 0.05, **p < 0.01, ***p < 0.001.

ROC analysis showed that a CRP level exceeding 4.3 mg/L predicted GIC with a sensitivity of 72.41% and a specificity of 73.63% (Area Under Curve 0.765, 95% CI 0.689–0.830, *p* < 0.001). Pearson correlation analysis showed a statistically significant moderate positive correlation between CRP and body mass index (BMI) (*r* = 0.302, *p* < 0.001). Additionally, multiple logistic regression analysis revealed that being a primary school graduate, along with higher BDNF and CRP levels, significantly influenced the likelihood of cognitive impairment (odds ratio 15.928, *p* < 0.001; odds ratio 1.063, *p* < 0.001; and odds ratio 1.260, *p* = 0.006, respectively) (Table 6).

**Table 6:** Univariate and multivariate regression analyses for cognitive impairment

	Univariate			Multivariate		
	OR	95% CI	*P*	OR	95% CI	*P*
Male gender	1.243	0.631–2.448	0.529			
Education (primary school)	12.343	4.516–33.735	<0.001***	15.928	5.057–50.169	<0.001***
Unemployed	2.115	1.050–4.260	0.036*	2.179	0.901–5.270	0.084
BMI	1.093	1.020–1.172	0.011*	1.012	0.927–1.105	0.785
Number of manic episodes	1.153	1.009–1.317	0.036*	1.062	0.911–1.239	0.441
Number of depressive episodes	1.019	0.902–1.150	0.764			
BDNF levels	1.037	1.013–1.061	0.003**	1.063	1.029–1.099	<0.001***
CRP levels	1.181	1.044–1.335	0.008**	1.260	1.068–1.486	0.006**
TSH levels	1.114	0.940–1.320	0.213			
SSRI use	0.279	0.078–1.000	0.060			

Logistic regression analyses.

OR, odds ratio; BMI, body mass index; BDNF, brain-derived neurotrophic factor; CRP, C-reactive protein; STSH, thyroid-stimulating hormone; SRI, selective serotonin reuptake inhibitor.

*p < 0.05, **p < 0.01, ***p < 0.001.

## Discussion

To the best of our knowledge, this study represents the first to explore serum levels of BDNF and CRP across distinct cognitive subgroups in individuals with bipolar disorder. This study provides critical insights into the cognitive heterogeneity observed in bipolar disorder by identifying three distinct cognitive subgroups: intact cognition, SIC and GIC. These findings contribute to the growing body of literature that highlights the variability in cognitive deficits among individuals with bipolar disorder, supporting the notion that bipolar disorder encompasses a broad spectrum of cognitive impairments rather than a uniform decline across all individuals.^28,29^ However, we recognise that external replication in independent cohorts would be important to further validate the generalisability of these subgroups. This cognitive variability has significant implications for personalised treatment approaches, as different subgroups may require tailored interventions targeting specific cognitive domains.

The significant difference in lithium levels observed among the cognitive subgroups provides important insights into the potential role of lithium in cognitive functioning in bipolar disorder. The finding that the GIC group had the highest lithium levels, while the intact cognition group had the lowest, suggests that higher lithium levels may be associated with greater cognitive impairment. This aligns with previous research indicating that while lithium is effective in stabilising mood and preventing relapse in bipolar disorder, its use at higher doses may contribute to cognitive side effects such as slowed processing speed, memory difficulties and executive dysfunction.^30–32^ The lack of significant differences between the SIC group and both the intact cognition and GIC groups further indicates that the relationship between lithium levels and cognitive performance may be most pronounced in cases of severe cognitive impairment. These findings underscore the need for careful lithium monitoring and individualised treatment plans that balance mood stabilisation with the potential for cognitive side effects, particularly in patients with severe cognitive deficits.

However, these findings should be interpreted with caution, as a substantial body of research suggests that lithium may have a neutral or positive impact on cognitive functioning. Cross-sectional and longitudinal studies of patients with bipolar disorder have found no significant impairments across key cognitive domains associated with lithium use.^33^ These findings, consistent with earlier research, suggest that lithium does not negatively impact cognition and may even offer neuroprotective benefits, despite frequent subjective reports of cognitive dulling. A recent systematic review of interventional studies on low-dose lithium reported positive effects in approximately half of the included trials, with the most consistent benefits observed for cognitive outcomes, particularly in Alzheimer’s disease and mild cognitive impairment. Although methodological heterogeneity and risk of bias limited the overall strength of evidence, the review emphasises the potential of low-dose lithium as a well-tolerated adjunct, especially where standard-dose treatment may not be suitable.^34^ The existing literature offers robust evidence indicating that lithium’s effects on cognition are complex and may not always result in impairment. As such, while our results point towards an association between higher lithium levels and greater cognitive decline, it is essential to consider the broader context of research, which highlights the variability in lithium’s cognitive effects depending on dosage, duration of treatment and individual patient factors. Future research should explore the mechanistic links between lithium exposure, dosage and specific cognitive domains to better inform clinical practice and optimise treatment outcomes in bipolar disorder.

Although recent research suggests a negative effect of valproate on cognitive functioning in chronically treated patients with bipolar disorder, particularly with working memory being the most affected domain,^35^ our study did not find significant differences between cognitive subgroups in terms of total valproate use duration or valproate serum levels. These findings suggest that, within our sample, valproate treatment may not have substantially contributed to the observed cognitive differences, although longer-term effects of valproate, interactions with other medications, illness severity, or individual biological and lifestyle factors cannot be entirely ruled out.

One of the key findings of this study is the association between serum levels of BDNF and cognitive performance. BDNF plays a crucial role in synaptic plasticity and neurogenesis, which are essential for maintaining cognitive function.^36^ The observation that higher BDNF levels were found in the SIC group suggests a potential protective or compensatory role of BDNF in mitigating cognitive decline in individuals with selective impairments. This aligns with recent studies that have reported positive correlations between BDNF levels and cognitive function in mood disorders, including bipolar disorder.^10^ However, the lower BDNF levels observed in the GIC group, characterised by widespread cognitive deficits, suggest that insufficient neuroplasticity may contribute to more severe cognitive impairments.^37^ These findings support the hypothesis that BDNF may serve as a biomarker for cognitive resilience in bipolar disorder,^38^ and interventions aimed at enhancing BDNF levels could offer therapeutic benefits for individuals at risk of cognitive decline. Biologically, however, elevated peripheral BDNF might also represent a compensatory upregulation in response to underlying neural stress, inflammation or cognitive dysfunction, rather than being directly protective. Methodologically, peripheral BDNF measures do not always mirror central nervous system levels and can be influenced by factors such as platelet release, circadian variation and medication effects. Finally, the cross-sectional design precludes determination of causality, and reverse causality cannot be ruled out, highlighting the need for longitudinal studies to clarify the direction of these associations.

Conversely, the relationship between elevated CRP levels and poorer cognitive performance, particularly in the GIC group, underscores the potential role of systemic inflammation in the pathophysiology of cognitive decline in bipolar disorder. Previous research has established a link between elevated inflammatory markers, such as CRP, and cognitive impairments in both psychiatric and non-psychiatric populations.^39–41^ In a large cohort study involving 222 euthymic bipolar disorder patients and 52 healthy controls, participants with CRP levels ≥5 mg/L showed significantly worse performance on tasks measuring executive functioning, processing speed, reasoning and problem-solving compared with those with lower CRP levels.^42^ The findings of this study are consistent with this body of research, demonstrating that higher CRP levels are associated with greater impairments in cognitive function. The identification of a CRP threshold that predicts GIC adds clinical value, as it provides a potential biomarker for identifying individuals at high risk for cognitive decline and guiding therapeutic interventions aimed at reducing inflammation.

While these findings reinforce the potential role of CRP in cognitive dysfunction, it is also important to consider metabolic factors that may influence systemic inflammation. In this context, the relationship between CRP and BMI warrants further examination.

Although our findings demonstrated a statistically significant correlation between CRP and BMI (*r* = 0.302, *p* < 0.001), the strength of this association was only moderate. This appears to contrast with previous research reporting a strong positive relationship between BMI and systemic inflammatory markers such as CRP, particularly in psychiatric populations.^43,44^ A study found that increased BMI was closely associated with elevated CRP levels in individuals with bipolar disorder, suggesting that metabolic dysregulation may play a substantial role in driving systemic inflammation.^45^ One plausible explanation for the relatively weaker correlation in our sample could be the limited sample size, which may have restricted the variability in BMI and CRP values and reduced the statistical power to detect stronger associations. Additionally, unmeasured confounding factors such as diet, physical activity, smoking status and use of medications with metabolic effects may have contributed to the attenuation of this relationship. Taken together, these findings highlight the need for future studies with larger and more heterogeneous samples to further elucidate the link between metabolic factors and systemic inflammation in bipolar disorder.

The distinct biological profiles observed in the SIC and GIC groups – characterised by differences in BDNF and CRP levels – highlight the complexity of the mechanisms underlying cognitive impairments in bipolar disorder. These findings suggest that cognitive impairments in bipolar disorder are driven by both neuroplasticity deficits and inflammation, with the balance between these factors determining the severity of cognitive decline. Future research should explore the interaction between these mechanisms, particularly the extent to which neuroinflammation affects neuroplasticity and contributes to cognitive deficits over time.

The implications of these findings for clinical practice are significant. First, the identification of cognitive subgroups in bipolar disorder underscores the need for personalised treatment approaches that address the specific cognitive deficits present in each group. Cognitive remediation programmes, which have been shown to improve cognitive outcomes in mood disorders,^46^ may be particularly effective for individuals in the SIC and GIC groups, where targeted interventions can address deficits in executive function, memory and processing speed. Additionally, interventions aimed at reducing systemic inflammation, such as anti-inflammatory medications or lifestyle interventions (e.g. exercise and dietary modifications), could be explored as potential strategies for mitigating cognitive decline in bipolar disorder patients with elevated CRP levels.

Furthermore, the association between BDNF levels and cognitive performance suggests that interventions designed to enhance neuroplasticity could improve cognitive outcomes in bipolar disorder. Physical exercise, which has been shown to increase BDNF levels, may represent a non-pharmacological strategy for improving cognitive function in individuals with SICs^47^ Pharmacological agents that enhance BDNF expression could also be explored as potential treatments for cognitive decline in bipolar disorder. These interventions, combined with regular cognitive assessments, could help prevent the progression of cognitive deficits and improve the overall quality of life for individuals with bipolar disorder.

However, recent systematic reviews and meta-analyses in mood disorders and neurodegenerative diseases emphasise both the variability and methodological challenges of using peripheral BDNF and CRP for biomarker-informed stratification. For example, while CRP has been associated with structural brain changes in mood disorders and BDNF alterations have been reported in depression and Alzheimer’s disease, confounding factors and heterogeneity limit the consistency and reliability of these markers as stratification tools.^48–50^ Nonetheless, both CRP and BDNF are modifiable through behavioural and pharmacological interventions, supporting the potential of integrating adjunctive strategies – such as exercise, dietary modification or anti-inflammatory treatments – into cognitive remediation programmes.^47,51^ Finally, longitudinal studies are warranted to determine whether biomarker-informed subgrouping can predict treatment response, thereby advancing the development of precision psychiatry approaches in bipolar disorder.

The study controlled for several confounding factors, including age, gender and medication use, specifically lithium, valproate, carbamazepine, antiepileptics such as gabapentin, pregabalin, topiramate, typical and atypical antipsychotics, SSRIs and benzodiazepines. None of the participants were taking medications that could potentially impair cognition, such as carbamazepine, gabapentin, pregabalin, topiramate or benzodiazepines, and the only antidepressants used were SSRIs. This reduces the likelihood that medication-related effects influenced the findings.

Despite the strengths of this study, including the use of a well-characterised sample and comprehensive cognitive assessments, several limitations should be acknowledged. The cross-sectional design limits the ability to draw conclusions about the causal relationships between biomarker levels and cognitive impairments. Longitudinal studies are needed to determine whether changes in BDNF and CRP levels over time are predictive of cognitive decline in bipolar disorder, as this would clarify temporal relationships and strengthen causal inference. We recognise this as a crucial next step and note that efforts are already underway to establish follow-up assessments in future studies.

Other factors that may influence cognitive performance, such as sleep disturbances and cardiovascular comorbid conditions (except stroke), were not accounted for. We also acknowledge the lack of assessment regarding the impact of polypharmacy, including the total number of psychotropic medications taken by participants, which could have influenced the results. Although patients with a formal anxiety disorder were excluded, individual subthreshold anxiety symptoms were not assessed, which may have affected cognitive performance in these participants.

Furthermore, certain variables that are known to affect serum BDNF levels, such as exercise, gonadal hormones and medication usage, were not explored in our investigation. It is also worth noting that CRP levels can fluctuate due to various illnesses, including asymptomatic viral infections. Although thyroid function was assessed through measurement of TSH levels, participants did not undergo broader medical screening procedures such as comprehensive metabolic panels or other systemic evaluations, limiting the ability to rule out potential medical comorbidities that might have influenced the findings. Information regarding thyroid hormone replacement therapy was also not available, which could have had an impact on both biomarker levels and cognitive outcomes.

Lithium levels were obtained at the same time as cognitive testing; however, trough and peak levels were not known, which limits the interpretation of potential lithium-related effects on cognitive performance and biomarker measurements. Moreover, no neuroimaging (MRI or computed tomography scan) or EEG data were available or reviewed for the participants, precluding further exploration of possible structural or electrophysiological correlates related to the cognitive or clinical findings.

Future studies should address these limitations and explore the role of additional biological markers, medical variables and environmental factors in contributing to cognitive impairments in bipolar disorder.

## Data Availability

The data that support the findings of this study are available from the corresponding author, A.M.D., upon reasonable request.
